# Experimental Investigations of Ni–Ti–Ru System: Liquidus Surface Projection and 1150 °C Isothermal Section

**DOI:** 10.3390/ma16155299

**Published:** 2023-07-27

**Authors:** Dupei Ma, Zhi Li, Yan Liu, Manxiu Zhao, Jingxian Hu

**Affiliations:** 1 School of Materials Science and Engineering, Xiangtan University, Xiangtan 411105, China; mdpdwyyx@163.com (D.M.); liuyan18773226132@163.com (Y.L.); zhaomanxiu@xtu.edu.cn (M.Z.); 2 Key Laboratory of Materials Design and Preparation Technology of Hunan Province, Xiangtan University, Xiangtan 411105, China

**Keywords:** Ni–Ti–Ru ternary system, phase equilibria, microstructure, X-ray diffraction

## Abstract

Ruthenium addition inhibits the formation of the topologically close-packed phases in Ni-based superalloys and improves the solid solution strength of Ni–Ti shape memory alloys. Therefore, the Ni–Ti–Ru phase stability is a very valuable indicator of the effects of Ru in Ni-based superalloys and Ni–Ti shape memory alloys. In this study, the isothermal section at 1150 °C and liquidus surface projection of the Ni–Ti–Ru ternary system were determined experimentally using the equilibrated alloy method and diffusion couple method, respectively. Alloys were prepared through the arc-melting of Ni, Ti, and Ru (all 99.99% purity), and then vacuum encapsulation in quartz tubes, followed by annealing at 1150 °C for 36 to 1080 h depending on the alloy composition. Diffusion couples were fabricated by joining one single-phase block (τ1) with one two-phase block (Ni_3_Ti + γ(Ni)), and the couples were annealed under vacuum at 1150 °C for 168 h. Reaction temperatures of as-cast alloys were determined by differential scanning calorimetry performed with heating and cooling rates of 10 °C/min. Scanning electron microscopy and X-ray diffraction were used to analyze the microstructure. Seven three-phase regions were found at the 1150 °C isothermal section. Seven primary solidification regions and five ternary invariant reactions were deduced in the liquidus surface projection. A new ternary compound τ1 was discovered in both the isothermal section at 1150 °C and liquidus surface projection. The results aid in thermodynamic modeling of the system and provide guidance for designing Ni-based superalloys and Ni–Ti shape memory alloys.

## 1. Introduction

Ni-based superalloys have been widely used as important components in aerospace engines and various gas turbines because of their good oxidation resistance and excellent mechanical properties at elevated temperatures. The exceptional high-temperature properties of Ni-based superalloys can be attributed to microstructural strengthening. The addition of Ti at low alloying concentrations will cause it to preferentially partition to the γ′ phase and serve to increase the antiphase boundary energy and strengthen the precipitate’s resistance to deformation [1,2,3]. A systematic investigation was carried out by Mishima et al. [4,5] on the temperature dependence of strength in the Ni_3_Al phase with the addition of Ti. The results indicated that as the concentration of Ti increased, residual levels of γ′ forming solutes may remain within the γ phase and subsequently serve as potent solid solution strengtheners. Then, Christofidou et al. [6] studied the microstructures and mechanical properties of multi-component Ni-based alloys and found that the addition of Ti can promote the formation of the γ and γ′ phases in Ni-based alloys. Further, the effect of the Ru addition on the microstructures and various mechanical properties of Ni-based alloys has been studied by many researchers [7,8,9,10]. Song et al. [7] found that a small amount of Ru is beneficial in impeding the formation of the topologically close-packed (TCP) phases and improving the creep resistance at high temperatures. However, Chen et al. [8,9] found that an excessive amount of Ru increased the degree of Re and Cr supersaturation in the γ phase and resulted in the TCP phase formation at high temperatures. More recently, Wang et al. [10] indicated that the variation of the Ru local atomic structure can greatly influence the microstructural evaluation and deformation mechanism by changing the lattice misfit and the vacancy formation energy, thus affecting the creep properties of Ni-based alloys.

Ni–Ti shape memory alloys (SMAs) are practically important functional materials because of their excellent shape memory effect, pseudo elasticity, and corrosion resistance [11,12]. The effects of Ru substitution on constituent phases, phase transformation temperatures, and mechanical properties were investigated for Ni–Ti SMAs by Tsuji et al. [13]. The results indicate that the addition of Ru results in solid solution strengthening by Ru substitution for the Ni sites in NiTi. With increasing Ru content, the lattice parameter of the B2 phase increases, the martensitic transformation temperature slightly decreases, and the melting temperature increases continuously. In addition, the Vickers hardness was maximum at an intermediate composition (HV1030 at 30 mol% Ru); this suggests that substantial solid solution hardening is caused by Ru substitution for the Ni sites in NiTi. Therefore, a change in the alloy composition affects the microstructure and phase equilibrium, which in turn affects the various mechanical properties of the alloy [14].

The phase diagram is like a map that provides a general guideline for designing the alloy compositions and the route of heat treatment. Therefore, the investigation of the phase diagram for the Ni–Ti–Ru ternary system is necessary for Ni-based superalloys and Ni–Ti SMAs.

In the present work, the diffusion couples, 15 annealed alloys, and 31 as-cast alloys were prepared to investigate the isothermal section at 1150 °C and liquidus surface projection. The chemical compositions of 15 annealed alloys and 31 as-cast alloys are listed in Table 1 and Table 2, respectively. The phases of the alloys were determined by scanning electron microscopy (SEM) combined with X-ray diffraction (XRD), and the composition of each phase in the alloys was measured by energy-dispersive spectroscopy (EDS). The reaction temperatures were determined using a differential scanning calorimeter (DSC). The experimental data obtained from the above three techniques were analyzed and are shown in Table 1, Table 2 and Table 3, respectively.

## 2. Literature Review

### 2.1. Binary Systems

**Figure 1:**
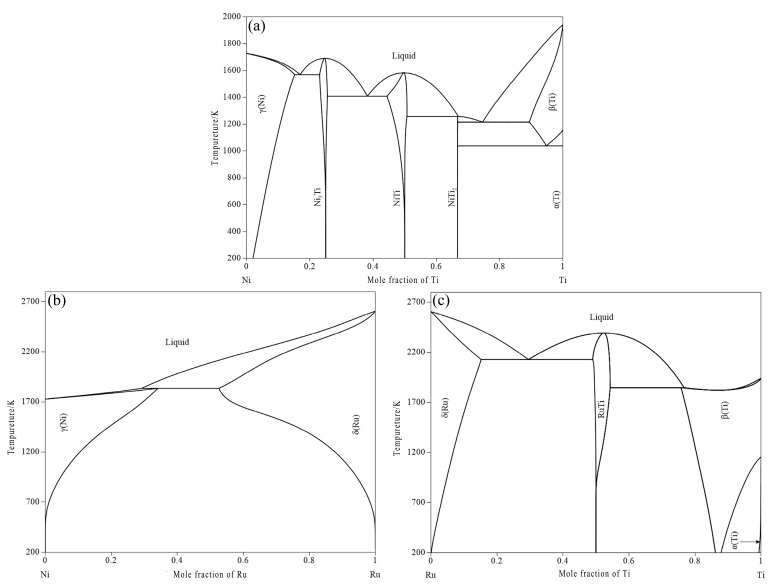
Calculated binary phase diagrams. (**a**) Ni–Ti system presented by Keyzer et al. [29]; (**b**) Ni–Ru system presented by Hallström [32]; (**c**) Ru–Ti system presented by Gao et al. [38].

The phase relationship of the Ni–Ti binary system has been studied systematically [16,17,18,19,20,21,22,23]. This system was assessed by several researchers on the basis of experimental data [24,25,26,27,28,29,30,31]. In recent times, the thermodynamic database of the Ni–Ti binary system built by Keyzer et al. [29] has been accepted, and the calculated phase diagram of the Ni–Ti binary system is shown in Figure 1a.

The Ni–Ru binary system is quite simple: Hallström [32] used the sub-regular solution model to re-evaluate this system, and the calculation result is shown in Figure 1b.

The Ru–Ti binary system was determined by Raub and Roeschel [33], Eremenko et al. [34], and Boriskina and Kornilov [35]. By using the experimental data presented by Eremenko et al. [24], Kaufman and Bernstein [36] and Mazhuga et al. [37] assessed this system in which the RuTi phase was treated as a kind of stoichiometric compound. Then, the Ru–Ti binary system was re-assessed by Gao et al. [38] based on the experimental data [33,34,35]. The solution phases were modeled with the Redlich–Kister equation and Kaptay equation. Then, the compound RuTi was described by a two-sublattice model (Ru,Ti)0.5(Ru,Ti)0.5. Figure 1c shows the evaluated Ru–Ti phase diagram.

### 2.2. Ni–Ti–Ru Ternary System

The Ni–Ti–Ru ternary system has been investigated in a few studies [15,39,40,41]. NiTi and RuTi with a CsCl-type structure are both isostructural phases, and their lattice parameters are also very similar. However, Boriskina [39] found a two-phase region between NiTi and RuTi, and they determined that the following peritectic reaction occurred at 1570 °C: Liq. (liquid phase) + RuTi ↔ NiTi. Eremenko et al. [40] doubted these results because the alloys were annealed at 1200 °C for only 25 h, making it difficult for the alloys to reach phase equilibrium; the authors suggested that there was an isomorphous-type pseudo binary system with a possible phase separation at lower temperatures. To verify the hypothesis, Semenova et al. [41] re-studied the NiTi–RuTi system by using the equilibrium alloy method [41] and differential thermal analysis (DTA). The results indicated that the NiTi–RuTi system was a simple isomorphous system, and a reasonably wide gap existed between the liquidus and solidus lines. In this case, the alloys had dendritic structures easily produced during solidification after smelting, and because the removal of this dendritic structure is difficult within only 25 h of annealing, the alloys in the earlier study [39] possibly appeared to have a two-phase equilibrium. Since the investigation of Semenova et al. [41] was not carried out below 1200 °C, it is not yet certain whether phase separation will occur in the NiTi–RuTi system at low temperatures.

On the basis of the results of the abovementioned studies, Velikanova et al. [15] reported a solidus surface projection and a part of a liquidus surface projection of the Ni–Ti–Ru ternary system. The research results showed that there were two perieutectic reaction points, U1 and U2, with the following perieutectic reactions: Liq. + γ(Ni) ↔ (Ni,Ru)Ti + Ni_3_Ti and Liq. + (Ni,Ru)Ti ↔ NiTi_2_ + β(Ti). However, the reaction types among Liq., (Ni,Ru)Ti, γ(Ni), and δ(Ru) were not determined. The crystallographic data for all phases related to the Ni–Ti–Ru system are listed in Table 4 [42,43,44,45,46,47,48,49].

**Table 4:** Crystallographic data of all phases related to Ni–Ti–Ru ternary system.

Phase	Strukturbericht Designation	Pearson Symbol	Space Group	Prototype	Reference
Liquid	/	/	/	/	/
γ(Ni)	*A*1	*cF*4	$Fm\bar{3}m$	Cu	[42]
β(Ti)	*A*2	*cI*2	$Im\bar{3}m$	W	[43]
α(Ti)	*A*3	*hP*2	$P6_{3}mmc$	Mg	[44]
δ(Ru)	*A*3	*hP*2	$P6_{3}mmc$	Mg	[45]
Ni_3_Ti	*D*0_24_	*hP*16	$P6_{3}mmc$	Ni_3_Ti	[46]
NiTi	*B*2	*cP*2	$Pm\bar{3}m$	CsCl	[47]
NiTi_2_	*E*9_3_	*cF*96	$Fd\bar{3}m$	NiTi_2_	[48]
RuTi	*B*2	*cP*2	$Pm\bar{3}m$	CsCl	[49]

## 3. Experimental Methods

The phase relationships in the Ni–Ti–Ru ternary system at 1150 °C were experimentally determined by using the equilibrated alloy method [41] and diffusion couples method [50]. Samples were prepared from 0.5 g each of 99.99% pure Ni particles, Ti particles, and Ru blocks. Each sample was accurately weighed with an electronic balance and determined to be 0.5 g in total. The alloys were prepared by arc melting with flowing argon on a water-cooled copper crucible and remelted at least three times to promote homogeneity. As Ti in the alloy is prone to oxidation which results in alloy composition deviation, pure Ti was smelted once before smelting the alloy to eliminate the possible residual oxygen in the melting furnace.

As-cast alloys were prepared to study the liquidus surface projection. The reaction temperatures of the as-cast alloys were determined by 404F3 DSC with Al_2_O_3_ crucibles and with heating and cooling rates of 10 °C/min.

The alloy specimens for the isothermal section were vacuum encapsulated in quartz tubes. The B1 and B2 alloys containing the liquid phase were annealed at 1150 °C for 36 h, while the other alloys were annealed for 1080 h. Finally, the sample capsules were quenched in water at 0 °C to retain the equilibria microstructures at 1150 °C.

The diffusion couples were fabricated across the Ni–Ti–Ru ternary system at 1150 °C. The single-phase alloy (τ1) and two-phase alloy (Ni_3_Ti + γ(Ni)) were sectioned into blocks to fabricate the diffusion couples. Each block was approximately 4 mm, 4 mm, and 3 mm in length, width, and thickness, respectively. The diffusion couples were vacuum encapsulated in quartz tubes and annealed at 1150 °C for 168 h.

All the samples were carefully ground and polished using standard metallographic preparation methods, and the 0.25 mm Al_2_O_3_ paste was used in the final polishing step. A chemical etching solution (10% HF + 20% HNO_3_ + 70% H_2_O) was used to reveal the microstructure of samples with no obvious atomic number contrast. The microstructure of each sample was examined by SEM (EVO MA10) with EDS to study the morphology and chemical composition of various phases in the samples. The acceleration voltage of 20 kV was applied. The chemical composition of each phase was measured at least thrice. After evaluation and the removal of unreliable data, the average values of the remaining reliable data were accepted and used for further analysis. To further confirm the phases of the samples, XRD analysis was performed using a Rigaku Ultimate IV operating at 100 mA and 40 kV with Cu Kα radiation. The diffraction spectra were collected from 20° to 100° with a scan step of 0.02°.

## 4. Results and Discussion

### 4.1. Isothermal Section at 1150 °C

**Figure 2:**
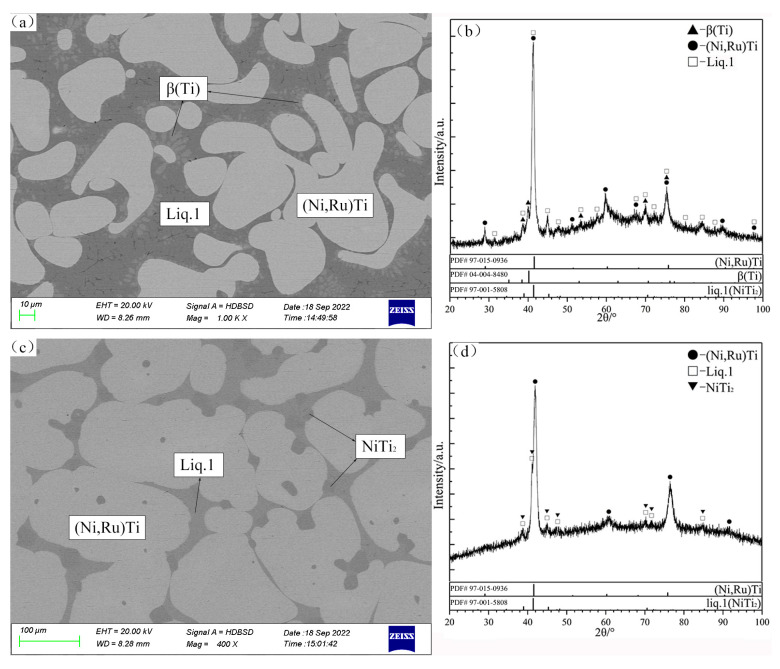
SEM micrographs (**a**,**c**) and XRD patterns (**b**,**d**) of annealed alloys B1 (**a**,**b**) and B2 (**c**,**d**).

**Figure 3:**
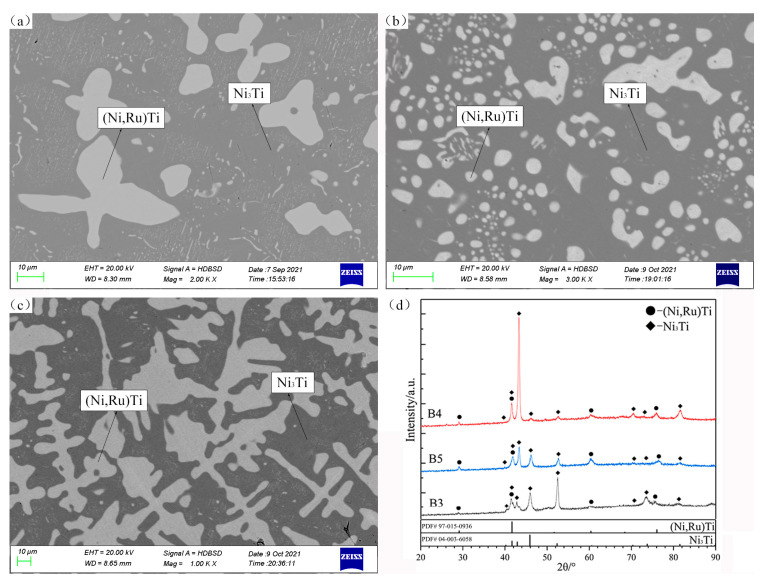
SEM micrographs (**a**–**c**) and XRD patterns (**d**) of annealed alloys B3 (**a**), B4 (**b**), and B5 (**c**).

**Figure 4:**
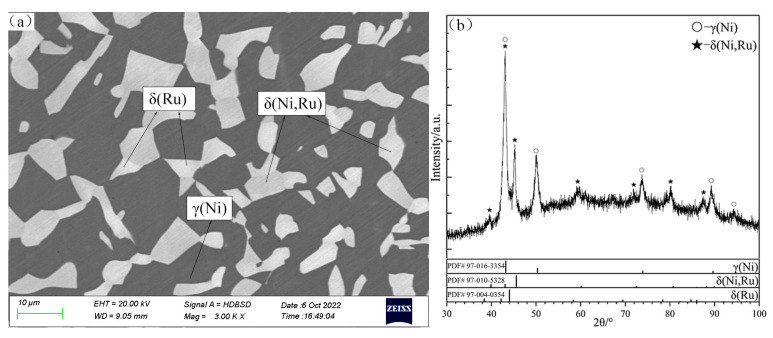
SEM micrograph (**a**) and XRD patterns (**b**) of annealed alloy B6.

**Figure 5:**
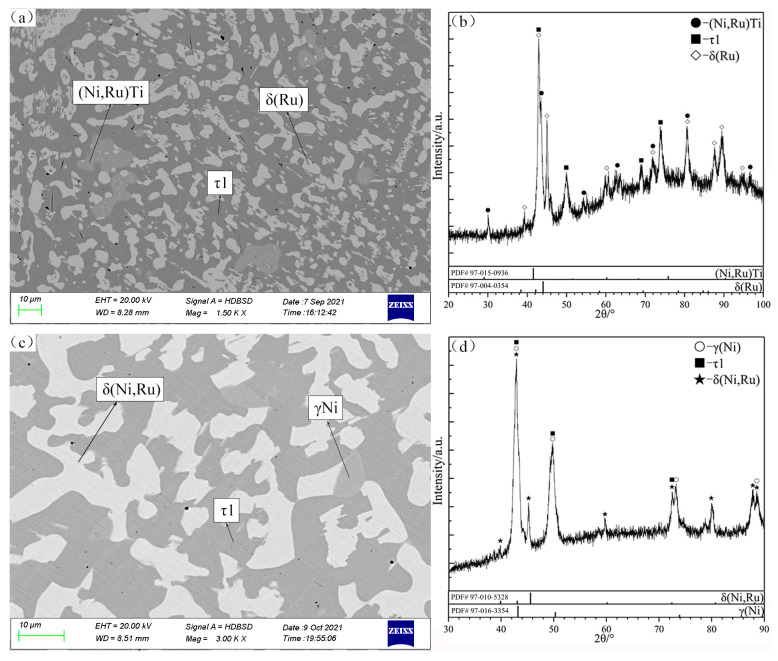
SEM micrographs (**a**,**c**) and XRD patterns (**b**,**d**) of annealed alloys B7 (**a**,**b**) and B8 (**c**,**d**).

**Figure 6:**
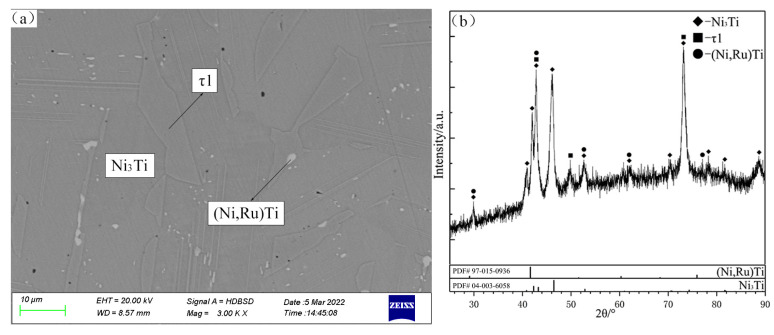
SEM micrograph (**a**) and XRD patterns (**b**) of annealed alloy B9.

**Figure 7:**
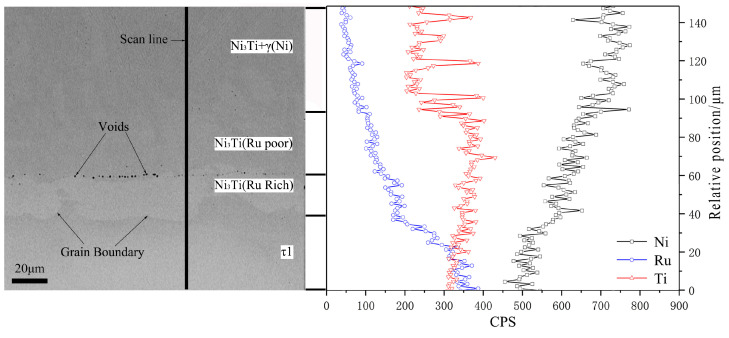
SEM micrograph and EDX composition profile of diffusion couples: (Ni_3_Ti + γ(Ni)) − τ1 at 1150 °C.

The constituent phases and compositions of annealing samples are listed in Table 1, and some typical SEM micrographs and XRD patterns are shown in Figure 2, Figure 3, Figure 4, Figure 5, Figure 6 and Figure 7.

As shown in the SEM micrographs of the alloys B1 and B2 (Figure 2a,c), the same typical perieutectic or peritectic microstructures are observed in the alloys, and the formation of these microstructures is due to the phase transformation of the liquid phase during the quenching process. The liquid phase in the alloy B1 is transformed into the NiTi_2_ phase that exists at low temperatures. Further, in agreement with the XRD patterns in Figure 2b, the three-phase equilibrium of Liq.1 + β(Ti) + (Ni,Ru)Ti can be identified, and the maximum Ru solubility of Liq.1 is 1.85 at.%. In the case of B2, a small amount of dark gray NiTi_2_ is also precipitated in the final stage of solidification. The two-phase region of Liq.1 + (Ni,Ru)Ti is determined with the SEM micrographs and XRD patterns in Figure 2c and Figure 2d, respectively.

In Figure 3, the SEM micrographs and XRD patterns of alloys B3, B4, and B5 confirm the presence of the two-phase region of (Ni,Ru)Ti + Ni_3_Ti. The two-phase regions of Liq.2 + NiTi and Liq.2 + Ni_3_Ti exist at 1150 °C according to the Ni–Ti binary system presented by Keyzer et al. [29]. Therefore, a narrow three-phase equilibrium region of (Ni,Ru)Ti + Ni_3_Ti + Liq.2 must be present in the Ni–Ti–Ru ternary system.

The SEM micrograph of the alloy B6 in Figure 4a shows a small δ(Ru) miscibility gap (marked as δ(Ru) and δ(Ni,Ru) in this isothermal section). Raub and Menzel [51] studied the Ni solubility of δ(Ru) in the Ni–Ru binary system at different temperatures corresponding to the crystal structure parameters of δ(Ni_x_,Ru_1−x_). The results [51] showed that with the increase in the temperature, the Ni solubility of δ(Ru) gradually increased and the lattice parameter “*a*” decreased from 2.691 to 2.612. Thus, the change in the lattice parameter will lead to a certain shift of X-ray characteristic peaks, which cannot be ignored in this study. The XRD patterns of the alloy B6 are shown in Figure 4b: the characteristic peaks of δ(Ni,Ru) are similar to those of δ(Ru) owing to the same HCP-A3 crystal structure, but the deviation is significant. In this study, the δ(Ru) phase is inferred to have undergone spinodal decomposition; in other words, δ(Ru) decomposed into two phases with the same crystal structure but different components. However, this hypothesis needs to be verified by performing more in-depth research. Due to the low content of the δ(Ru) phase in B6, the XRD patterns do not show the peaks of the δ(Ru) phase. In addition, it is uncertain whether the miscibility gap between δ(Ru) and δ(Ni,Ru) exists in the area with a higher Ti content.

There are two typical three-phase regions of (Ni,Ru)Ti + τ1 + δ(Ru) and γ(Ni) + τ1 + δ(Ni,Ru) observed in the alloys B7 and B8 (Figure 5a,c), which are in good agreement with XRD patterns (Figure 5b and Figure 5d, respectively). According to the SEM micrograph and XRD patterns shown in Figure 6a and Figure 6b, respectively, the Ni_3_Ti, (Ni,Ru)Ti and τ1 phases can be identified clearly in B9. The homogeneity ranges of the τ1 phase are determined to be from 11.06 to 28.19 at.% Ru and 19.69 to 25.14 at.% Ti.

The SEM micrograph and composition profiles of the τ1 and Ni_3_Ti + γ(Ni) diffusion couples are given in Figure 7. The total range of the interdiffusion zone is approximately 40–50 μm, and the Ni_3_Ti + γ (Ni) two-phase region gradually transforms into a thick layer Ni_3_Ti-(Ru-poor). Further, a continuous layer of Ni_3_Ti-(Ru-rich) of approximately 15–25 μm is formed between the Ni_3_Ti-(Ru-poor) and τ1. This shows that the flux of Ni atoms causes diffusion into the τ1 phase, which results in the formation of Ni_3_Ti-(Ru-poor) and Ni_3_Ti-(Ru-rich).

In Figure 7a, the grain boundary between Ni_3_Ti-(Ru-rich) and τ1 is not perfectly planar. Some Kirkendall voids appear at the boundary between Ni_3_Ti-(Ru-rich) and Ni_3_Ti-(Ru-poor) due to interdiffusion. Thus, the diffusion path between τ1 and Ni_3_Ti + γ(Ni) at 1150 °C is as follows: τ1 single-phase region, Ni_3_Ti single-phase region, and then the Ni_3_Ti + γ(Ni) two-phase region. From this result and the two-phase regions τ1 + γ(Ni) and Ni_3_Ti + γ(Ni) found in B14 and B15 alloys, respectively, a three-phase region τ1 + Ni_3_Ti + γ(Ni) can be inferred.

In addition, the B10 and B13 alloys support the two-phase regions of (Ni,Ru)Ti + τ1 and δ(Ni,Ru) + τ1, respectively. The B11 and B12 alloys are all located in the two-phase region of δ(Ni,Ru) + γ(Ni).

### 4.2. Liquidus Surface Projection

In this work, 31 as-cast alloys were prepared to determine the liquidus surface projection of the Ni–Ti–Ru ternary system, and the alloy compositions and primary solidification phases are listed in Table 2.

**Figure 8:**
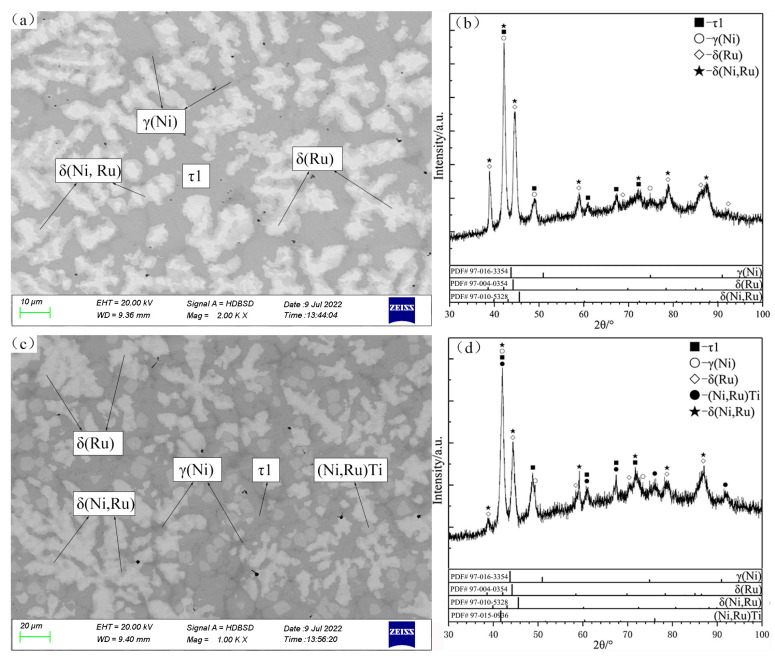
SEM micrographs (**a**,**c**) and XRD patterns (**b**,**d**) of as-cast alloys C3 (**a**,**b**) and C4 (**c**,**d**).

The alloys C3 and C4 are located in the primary solidification region δ(Ru) (Figure 8a,b). Notably, the δ(Ru) phase shows a miscibility gap due to the spinodal decomposition that is discussed in Section 4.1. According to the SEM micrograph and XRD patterns of the alloy C3, the gray phase surrounding δ(Ru) is identified as the τ1 phase that is generated by the peritectic reaction: liq. + δ(Ru) ↔ τ1. As shown in Figure 8c, in the case of C4, in addition to the phases of C3, a grayish-white phase exists, and it was determined to be the (Ni,Ru)Ti phase on the basis of the XRD patterns (Figure 8d).

**Figure 9:**
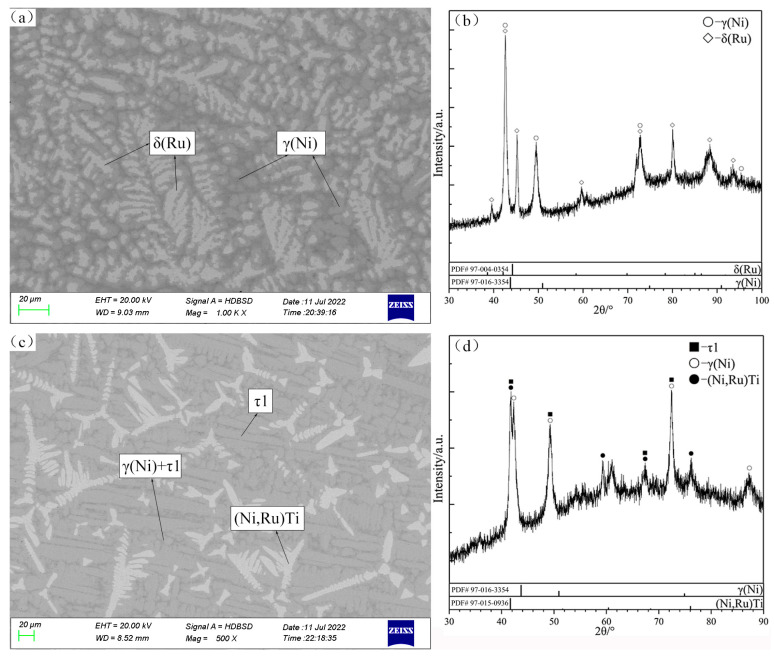
SEM micrographs (**a**,**c**) and XRD patterns (**b**,**d**) of as-cast alloys C6 (**a**,**b**) and C15 (**c**,**d**).

The alloy C6 is located in the primary solidification region δ(Ru) (Figure 9a,b), and this alloy does not undergo the expected perieutectic reaction: liq. + δ(Ru) ↔ τ1 + γ(Ni); instead, the remaining liquid phase solidifies into γ(Ni) in the peritectic reaction: liq. + δ(Ru) ↔ γ(Ni). According to the SEM micrograph and XRD patterns of the alloy C15 (Figure 9c,d), a large amount of the dendritic primary (Ni,Ru)Ti phase can be observed. The typical eutectic microstructure of this alloy is γ(Ni) + τ1, with the (Ni,Ru)Ti phase enclosed by τ1, which is formed by the peritectic reaction. In addition, some amount of lathy sub-primary solidification phase τ1 also exists.

**Figure 10:**
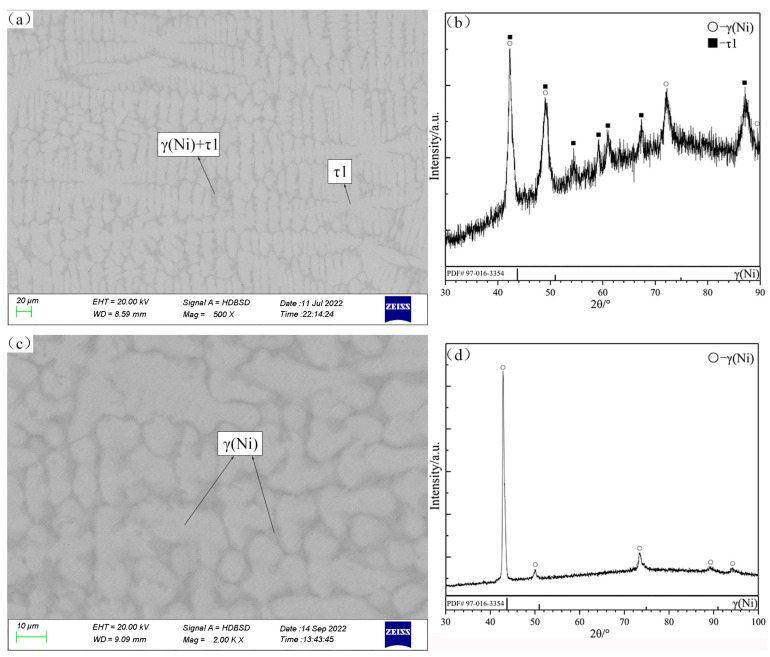
SEM micrographs (**a**,**c**) and XRD patterns (**b**,**d**) of as-cast alloys C14 (**a**,**b**) and C26 (**c**,**d**).

Among the series of alloys with the primary solidification phase τ1, the alloy C14 (Figure 10a,b) was considered for the subsequent analyses. Its SEM microstructure is similar to that of the alloy C15, except that the (Ni,Ru)Ti phase is missing. Therefore, the solidification path of the alloy C14 is simple: liq. ↔ τ1, liq. ↔ γ(Ni) + τ1. In addition, Figure 10c shows the primary phase γ(Ni) of the alloy C26 during solidification.

**Figure 11:**
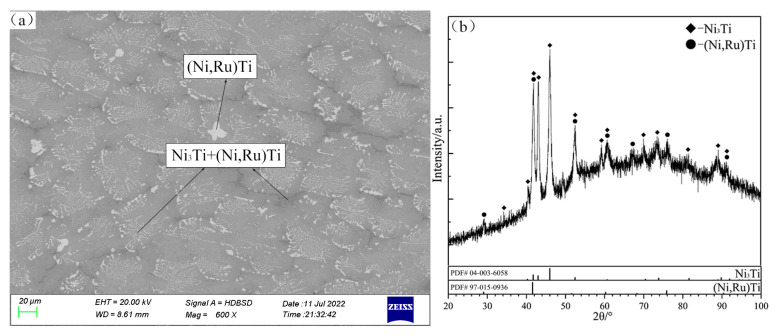
SEM micrograph (**a**) and XRD patterns (**b**) of as-cast alloy C10.

**Figure 12:**
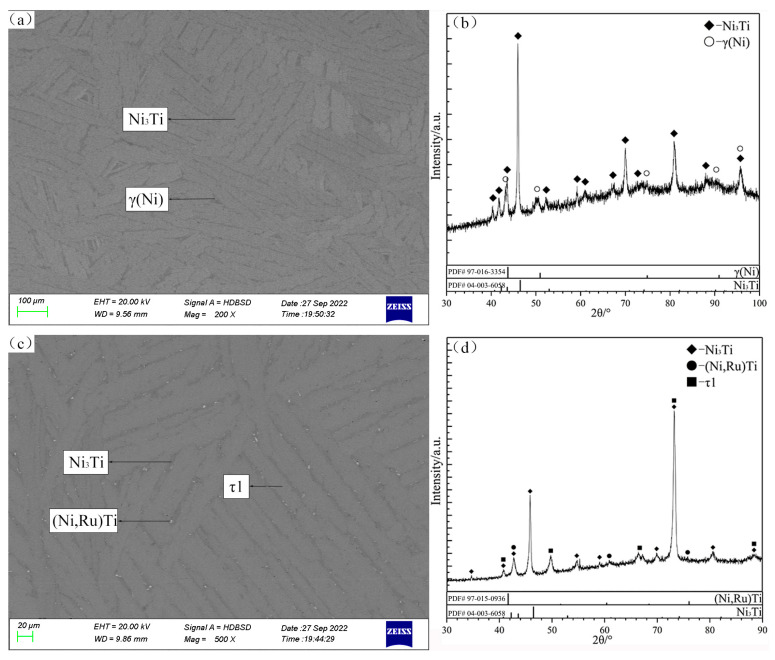
SEM micrographs (**a**,**c**) and XRD patterns (**b**,**d**) of as-cast alloys C30 (**a**,**b**) and C31 (**c**,**d**).

Based on the XRD patterns and SEM micrograph of the alloy C10 (Figure 11), a few primary solidification phases of (Ni,Ru)Ti and eutectic microstructure Ni_3_Ti + (Ni,Ru)Ti are identified. In the final stage of solidification, some amount of eutectic microstructure Ni_3_Ti + NiTi formed at the grain boundary, due to the binary eutectic reaction: liq. ↔ Ni_3_Ti + NiTi. The alloy C30 is located in the Ni_3_Ti primary solidification region, while the alloy C31 is located in the τ1 region. From the SEM micrograph and XRD patterns of alloy C30 shown in Figure 12a and Figure 12b, respectively, the light gray phase is determined to be the primary solidification phase Ni_3_Ti. The solidification path of alloy C30 is inferred to be as follows: liq. ↔ Ni_3_Ti, liq. ↔ Ni_3_Ti + γ(Ni). The SEM micrograph and XRD patterns of alloy C31 (Figure 12c and Figure 12d, respectively) confirm the existence of the τ1, (Ni,Ru)Ti, and Ni_3_Ti phases, and the solidification path of the alloy C31 is inferred to be as follows: liq. ↔ τ1, liq. + τ1 ↔ (Ni,Ru)Ti + Ni_3_Ti. A comparison of the difference in the paths between alloys C30 and C31 indicate that Ni_3_Ti and τ1 phases can be clearly distinguished and that the existence of a maximum point e*_max_* on the univariate line (liq. ↔ Ni_3_Ti + τ1) can be inferred.

**Figure 13:**
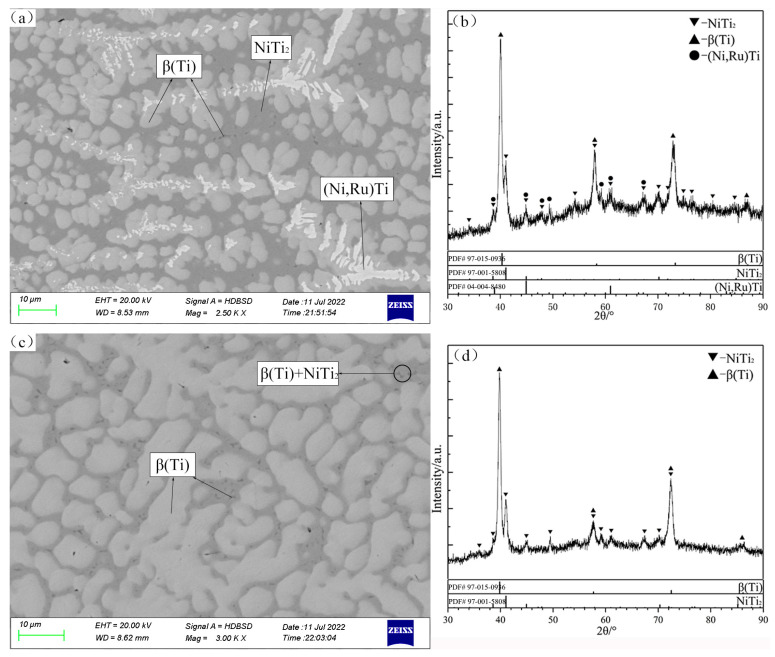
SEM micrographs (**a**,**c**) and XRD patterns (**b**,**d**) of as-cast alloys C12 (**a**,**b**) and C13 (**c**,**d**).

The SEM micrograph and XRD patterns of the alloy C12 are shown in Figure 13a and Figure 13b, respectively. The primary solidification phase of (Ni,Ru)Ti is observed in C12. Obviously, dendritic (Ni,Ru)Ti first precipitates from the liquid phase, and then the peritectic reaction liq. + (Ni,Ru)Ti ↔ β(Ti) occurs. Finally, the remaining liquid phase undergoes binary eutectic reaction liq. ↔ β(Ti) + NiTi_2_. The SEM micrograph and XRD patterns of the alloy C13 (Figure 13c and Figure 13d, respectively) show that its primary solidification phase is β(Ti). The eutectic microstructure of the alloy C13 is β(Ti) + NiTi_2_, which is formed by the same eutectic reaction liq. ↔ β(Ti) + NiTi_2_ (like alloy C12).

**Figure 14:**
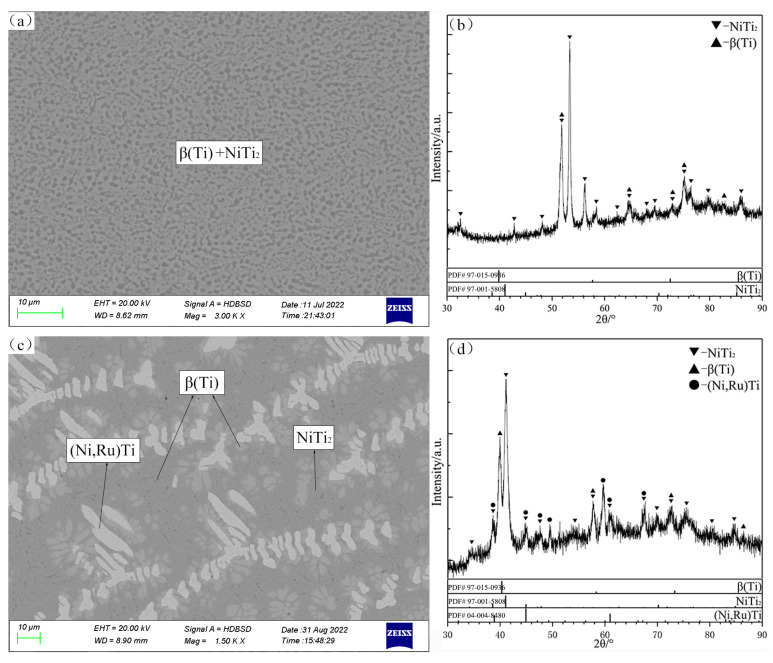
SEM micrographs (**a**,**c**) and XRD patterns (**b**,**d**) of as-cast alloys C11 (**a**,**b**) and C23 (**c**,**d**).

According to the SEM micrograph and XRD patterns of the alloy C11, shown in Figure 14a and Figure 14b, respectively, there is a typical eutectic microstructure β(Ti) + NiTi_2_. The SEM micrograph and XRD patterns of the alloy C23 are shown in Figure 14c and Figure 14d, respectively: a perieutectic reaction liq. + (Ni,Ru)Ti ↔ β(Ti) + NiTi_2_ and eutectic reaction liq. ↔ β(Ti) + NiTi_2_ occur in the alloy.

According to the above experimental results of the primary phases and solidification structures of the as-cast alloys, the solidification paths of the as-cast alloys not presented in the discussion so far are as follows:

Alloy C3: liq. ↔ δ(Ru), liq. + δ(Ru) ↔ τ1, liq. + δ(Ru) ↔ τ1 + γ(Ni), liq. ↔ τ1 + γ(Ni);

Alloy C4: liq. ↔ δ(Ru), liq. ↔ δ(Ru) + (Ni,Ru)Ti, liq. + (Ni,Ru)Ti + δ(Ru) ↔ τ1, liq. + δ(Ru) ↔ τ1, liq. + δ(Ru) ↔ τ1 + γ(Ni), liq. ↔ τ1 + γ(Ni);

Alloy C15: liq. ↔ (Ni,Ru)Ti, liq. + (Ni,Ru)Ti ↔ τ1, liq. ↔ τ1, liq. ↔ γ(Ni) + τ1;

Alloy C31: liq. ↔ τ1, liq. + τ1 ↔ (Ni,Ru)Ti + Ni3Ti;

Alloy C12: liq. ↔ (Ni,Ru)Ti, liq. + (Ni,Ru)Ti ↔ β(Ti), liq. ↔ β(Ti) + NiTi2;

Alloy C13: liq. ↔ β(Ti), liq. ↔ β(Ti) + NiTi2;

Alloy C23: liq. ↔ (Ni,Ru)Ti, liq. + (Ni,Ru)Ti ↔ β(Ti) + NiTi2, liq. ↔ β(Ti) + NiTi2.

**Figure 15:**
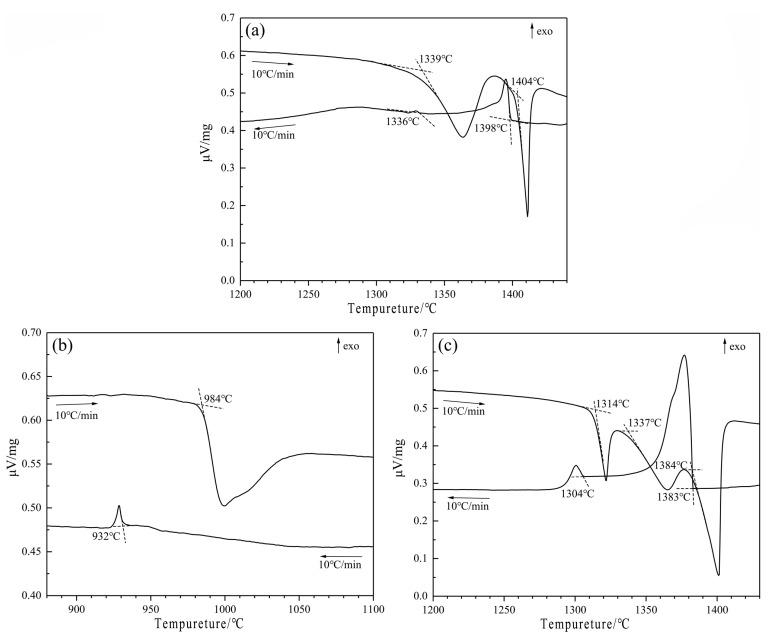
Results of DSC analyses of as-cast alloys: (**a**) C4; (**b**) C23; (**c**) C31.

Alloys C4, C23, and C31 are selected to measure the temperatures of the invariant reaction by DSC, and the DSC curves of the alloys are shown in Figure 15. Two peaks starting at 1404 °C on heating and 1398 °C on cooling in Figure 15a correspond to the reaction U1 (liq. + δ(Ru) ↔ τ1 + γ(Ni)). Further, two peaks starting at 1339 °C on heating and 1336 °C on cooling correspond to the eutectic reaction (liq. ↔ τ1 + γ(Ni)). Figure 15b shows the peak starting at 984 °C on heating of the alloy C23 which corresponds to the reaction U4 (liq. + (Ni,Ru)Ti ↔ β(Ti) + NiTi_2_). In addition, due to the volatilization of Ni at high temperatures, the composition of the C23 alloy changes and the eutectic reaction (liq. ↔ β(Ti) + NiTi_2_) occurs in the remaining liquid phase, corresponding to the peak starting at 932 °C on cooling. The peaks starting at 1384 °C and 1314 °C on heating and at 1383 °C and 1304 °C on cooling of the alloy C31 (Figure 15c) correspond to the three-phase eutectic reactions (liq. ↔ Ni_3_Ti + τ1) and (liq. ↔ (Ni,Ru)Ti + Ni_3_Ti), respectively. Furthermore, the reaction temperature of P1 and e_max_ are inferred as being about 1500 °C and 1390 °C, respectively. Notably, only the peak starting at 1337 °C on heating corresponding to the reaction U3 (liq. + τ1 ↔ (Ni,Ru)Ti + Ni_3_Ti) is found in the curves (Figure 15c). This may also be caused by the volatilization of the element. Further, it can be inferred that the reaction temperature of U2 should be about 1330 °C.

**Figure 16:**
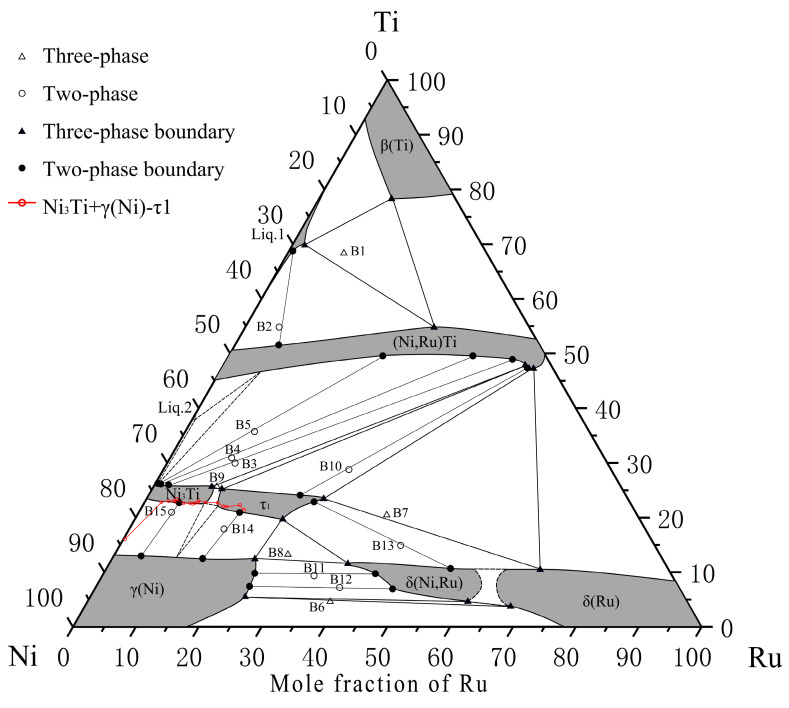
Isothermal section of Ni–Ti–Ru ternary system at 1150 °C.

**Figure 17:**
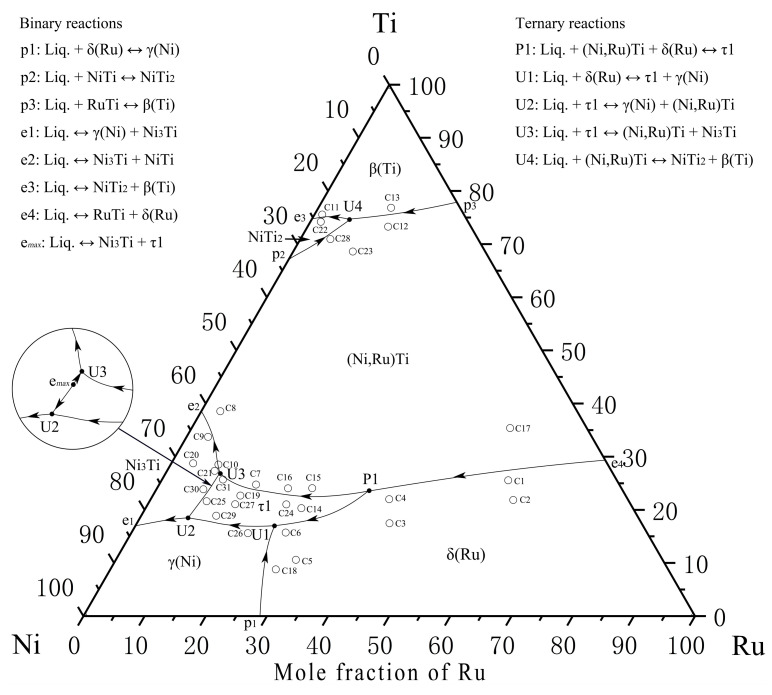
Liquidus surface projection of Ni–Ti–Ru ternary system.

**Figure 18:**
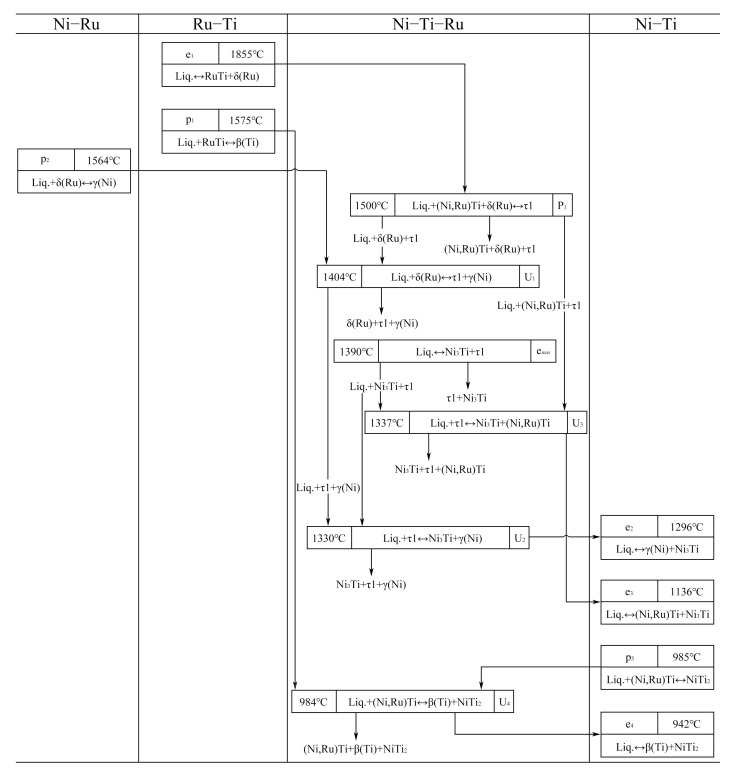
Invariant reaction scheme for Ni–Ti–Ru ternary system.

According to the above experimental results and discussion, the isothermal section of the Ni–Ti–Ru ternary system at 1150 °C is presented in Figure 16. The invariant reaction temperatures are shown in Table 3. The predicted liquidus surface projection and complete reaction scheme are shown in Figure 17 and Figure 18, respectively.

## 5. Conclusions

The isothermal section at 1150 °C and the liquidus surface projection of the Ni–Ti–Ru ternary system have been established by using SEM/EDS, XRD, and DSC analyses.

The isothermal section at 1150 °C has five determined three-phase regions, namely, Liq.1 + β(Ti) + (Ni,Ru)Ti, δ(Ru) + δ(Ni,Ru) + γ(Ni), (Ni,Ru)Ti + τ1 + δ(Ru), γ(Ni) + τ1 + δ(Ni,Ru), Ni_3_Ti + (Ni,Ru)Ti + τ1, and two speculated three-phase regions, namely, τ1 + Ni_3_Ti + γ(Ni) and (Ni,Ru)Ti + Ni_3_Ti + Liq.2.

The whole liquidus surface projection and related invariant reaction schemes are proposed, with seven primary solidification regions: β(Ti), NiTi_2_, (Ni,Ru)Ti, Ni_3_Ti, τ1, δ(Ru), and γ(Ni) and five invariant reactions: P1 (liq. + (Ni,Ru)Ti + δ(Ru) ↔ τ1), U1 (liq. + δ(Ru) ↔ τ1 + γ(Ni)), U2 (liq. + τ1 ↔ γ(Ni) + Ni_3_Ti), U3 (liq. + τ1 ↔ (Ni,Ru)Ti + Ni_3_Ti), and U4 (liq. + (Ni,Ru)Ti ↔ β(Ti) + NiTi_2_).

The addition of Ti seems to result in a small δ(Ru) miscibility gap due to spinodal decomposition. A new ternary compound τ1 is identified based on the present study on both the isothermal section and liquidus surface projection.

## Figures and Tables

**Table 1:** Constituent phases and compositions of samples annealed at 1150 °C isothermal section in Ni–Ti–Ru ternary system.

Sample	Nominal Composition/at.%	Phase	Composition/at.%		
			Ni	Ti	Ru
B1	Ni15.76Ti68.47Ru15.77	(Ni,Ru)Ti	15.04	55.00	29.96
		β(Ti)	10.16	78.17	11.67
		Liq.1	28.14	70.01	1.85
B2	Ni39.79Ti54.80Ru5.41	Liq.1	30.84	68.81	0.71
		(Ni,Ru)Ti	41.44	51.46	7.10
B3	Ni59.17Ti29.94Ru20.44	Ni_3_Ti	71.70	26.02	2.28
		(Ni,Ru)Ti	5.53	48.97	45.50
B4	Ni59.28Ti30.92Ru9.80	Ni_3_Ti	72.93	26.20	0.87
		(Ni,Ru)Ti	11.55	49.53	38.92
B5	Ni53.28Ti35.65Ru11.06	Ni_3_Ti	73.29	26.23	0.48
		(Ni,Ru)Ti	25.84	49.63	24.53
B6	Ni56.61Ti4.76Ru38.63	δ(Ru)	28.43	3.83	67.75
		δ(Ni,Ru)	34.86	4.61	60.53
		γ(Ni)	69.86	5.52	24.62
B7	Ni39.74Ti20.59Ru39.67	δ(Ru)	20.31	10.87	68.82
		τ1	48.37	23.44	28.19
		(Ni,Ru)Ti	2.94	47.43	49.63
B8	Ni59.08Ti13.36Ru27.56	δ(Ni,Ru)	50.44	11.63	37.93
		τ1	56.91	19.69	23.40
		γ(Ni)	63.49	12.27	22.24
B9	Ni64.51Ti25.59Ru9.90	τ1	63.80	25.14	11.06
		(Ni,Ru)Ti	4.04	47.80	48.16
		Ni3Ti	65.08	25.63	24.53
B10	Ni41.64Ti28.77Ru29.59	τ1	51.79	24.09	24.12
		(Ni,Ru)Ti	4.15	48.59	47.26
B11	Ni56.93Ti9.32Ru33.75	δ(Ni,Ru)	46.98	9.77	43.25
		γ(Ni)	66.09	9.79	24.12
B12	Ni54.50Ti5.30Ru40.20	δ(Ni,Ru)	45.60	7.02	47.38
		γ(Ni)	68.13	7.38	24.49
B13	Ni40.42Ti14.87Ru14.71	τ1	50.17	22.77	27.06
		δ(Ni,Ru)	34.62	10.71	54.67
B14	Ni67.01Ti17.84Ru15.15	τ1	62.99	20.96	16.05
		γ(Ni)	73.06	12.49	14.45
B15	Ni73.81Ti20.94Ru5.26	Ni_3_Ti	71.69	22.71	5.60
		γ(Ni)	82.55	13.15	4.30

**Table 2:** Alloy compositions and primary solidification phases of as-cast alloys.

Sample	Alloy Compositions/at.%			Primary Solidification Phase
	Ni	Ti	Ru	
C1	17.76	25.54	56.70	δ(Ru)
C2	18.80	21.85	59.36	δ(Ru)
C3	41.22	17.48	41.30	δ(Ru)
C4	39.00	22.01	38.99	δ(Ru)
C5	59.96	10.59	29.45	δ(Ru)
C6	59.06	15.72	25.22	δ(Ru)
C7	59.39	24.74	15.86	(Ni,Ru)Ti
C8	58.33	38.56	3.11	(Ni,Ru)Ti
C9	62.75	33.73	3.52	Ni_3_Ti
C10	63.68	28.50	7.82	Ni_3_Ti + (Ni,Ru)Ti
C11	23.17	75.57	1.26	β(Ti) + NiTi_2_
C12	13.54	73.27	13.19	(Ni,Ru)Ti
C13	11.27	76.83	11.90	β(Ti)
C14	54.21	20.31	25.48	τ1
C15	50.54	24.08	25.38	(Ni,Ru)Ti
C16	54.51	24.05	21.44	(Ni,Ru)Ti
C17	12.55	35.39	52.07	(Ni,Ru)Ti
C18	64.14	8.78	27.07	δ(Ru)
C19	62.99	22.67	13.34	τ1
C20	67.63	28.79	3.58	Ni_3_Ti
C21	64.88	27.32	7.80	Ni_3_Ti + (Ni,Ru)Ti
C22	27.07	74.20	1.72	β(Ti) + NiTi_2_
C23	21.65	68.60	9.75	(Ni,Ru)Ti
C24	56.31	21.01	22.68	τ1
C25	69.05	21.60	9.35	Ni_3_Ti + τ1
C26	65.29	15.61	19.10	γ(Ni)
C27	64.64	21.06	14.30	τ1
C28	24.14	70.94	4.92	(Ni,Ru)Ti
C29	68.83	18.90	12.26	τ1
C30	68.44	23.87	7.69	Ni_3_Ti + γ(Ni)
C31	64.33	25.69	9.98	τ1

**Table 3:** Invariant reactions in Ni–Ti–Ru ternary system.

Invariant Reaction	Reaction Type	Reaction Temperature (°C)	Reference
liq. + (Ni,Ru)Ti + δ(Ru)	P1 ^a^	~1500	This work
liq. + δ(Ru) ↔ τ1 + γ(Ni)	U1	1404	This work
liq. + τ1 ↔ γ(Ni) + Ni_3_Ti	U2 ^a^	~1330	This work
liq. + τ1 ↔ (Ni,Ru)Ti + Ni_3_Ti	U3	1337	This work
liq. + (Ni,Ru)Ti ↔ β(Ti) + NiTi_2_	U4	984	This work
		980	[15]

^a^ Note that the invariant reactions U1, U3, and U4 are determined directly, and P1 and U2 are inferred in this work.

## Data Availability

The data presented in this study are available on request from the corresponding author. The data are not publicly available because it is a part of an ongoing study.
